# Liraglutide attenuates atherosclerosis via inhibiting ER-induced macrophage derived microvesicles production in T2DM rats

**DOI:** 10.1186/s13098-017-0289-y

**Published:** 2017-12-01

**Authors:** Jinjin Li, Xiaojuan Liu, Qianhua Fang, Min Ding, Chunjun Li

**Affiliations:** Key Laboratory of Hormones and Development (Ministry of Health), Tianjin Key Laboratory of Metabolic Diseases, Tianjin Metabolic Diseases Hospital & Tianjin Institute of Endocrinology, Tianjin Medical University, No. 22, Qixiangtai Road, Tianjin, 300070 People’s Republic of China

**Keywords:** Diabetes, Atherosclerosis, Liraglutide, Macrophage, Microvesicle

## Abstract

**Background:**

We investigated the effects of liraglutide on the formation and progression of atherosclerosis in type 2 diabetes mellitus (T2DM) rats.

**Methods:**

Sprague–Dawley rats were divided into control group, diabetes group and liraglutide treated group. The T2DM rats model with atherosclerosis were induced by high fat diet followed small dosage streptozotocin injection. Body weight and blood glucose levels were monitored once a week for 3 months and then the rats were sacrificed.Peripheral blood and aorta tissues were collected for further biochemical and pathological estimation respectively. Moreover, immunohistochemistry staining was used to detect the infiltration of macrophages and cell apoptosis in tissue samples. The amount of microvesicles of atherosclerotic plaques was determined by ELISA. Western blot was applied to detect the protein expressions of CHOP, GRP78 and caspase-3 in tissue samples. The mRNA expressions of SREBP-1c and FAS were detected by RT-PCR.

**Results:**

The rat model of diabetic atherosclerosis was established successfully. Compared with the control group, glucose, triglycerides, total cholesterol, AST, ALT, BUN, fasting insulin and homeostatic model assessment insulin resistance levels in peripheral blood were significantly increased in the diabetes group. While, these indicators in the liraglutide group were significantly lower than that in the diabetes group. Moreover, the atherosclerotic plaques were observed in the rats of diabetes group but not remarkable in the liraglutide group. The ratio between aorta intima and media thickness was significantly greater in the diabetes group than that in the liraglutide group. Compared with the diabetes group, the infiltration and apoptosis of macrophages were milder in the liraglutide group. The expressions of CD68, caspase-3, CHOP and GRP78 in aorta tissue samples were significantly downregulated in the liraglutide group than that in the diabetes group. Furthermore, the microvesicles of aorta tissues in the liraglutide group were significantly decreased than that in the diabetes group. The mRNA expressions of SREBP-1c and FAS were lower in the liraglutide group than that in the diabetes group.

**Conclusion:**

Liraglutide attenuates diabetic atherosclerosis by inhibition of ER stress and subsequent macrophage apoptosis and microvesicles production in T2DM rats.

## Background

Despite the progress of therapy, the prevalence of type 2 diabetes mellitus (T2DM) is increasing and diabetes has become an important public health problem worldwide [1]. T2DM is known to promote the atherosclerotic process which predisposes to cardiovascular disorders. As the process continues, the narrowing of the vessel lumen occurs, leading to acute cardiovascular events [2]. Cardiovascular complications are the leading cause of diabetes-related morbidity and mortality [3]. Therefore, to elucidate the mechanism of diabetic atherosclerosis and to develop the new drugs are helpful to improve the prognosis of diabetic patients.

Endoplasmic reticulum (ER) stress is defined as an imbalance between client protein load and folding capacity which can potentially lead to ER dysfunction [4]. ER stress is a pathogenic mechanism associated with not only diabetes mellitus [5] but also various cardiovascular diseases, including coronary heart disease, cardiac ischemia–reperfusion injury and cardiomyopathy [6, 7]. Excessive ER stress can induce apoptosis of endothelial cells, macrophages and smooth muscle cells, which promotes the formation and development of atherosclerotic plaque [8]. Moreover, ER stress can also induce the shedding release of microvesicles (MVs) from endothelium [9]. MVs are small vesicles between 100 and 500 nm in all kinds of cells. MVs can promote the progress of atherosclerosis in the diabetic patients [10]. Therefore, apoptosis and MVs induced by ER stress contribute to the diabetic atherosclerosis.

Liraglutide is an analog of human glucagon-like peptide-1 (GLP-1) and has 97% amino acid homology with human GLP-1. GLP-1 is an endogenous incretin peptide hormone secreted from the gut, which plays a key physiological role in the blood glucose homeostasis [11]. Liraglutide is the GLP-1 receptor agonist and takes an glucose-lowering effect. Therefore, liraglutide is now used therapeutically in the diabetic patients as a new antidiabetic drug. In addition to downregulating the blood glucose, liraglutide can also take a protective effect in the type 2 diabetic patients who undergo the cardiovascular events [12]. Moreover, liraglutide can inhibit the ER stress in the diabetic cardiomyopathy [13]. Consequently, liraglutide potentially plays a pivotal role both in the metabolic and the cardiovascular system.

Above all, excessive ER stress can promote the progress of atherosclerosis by inducing apoptosis and MVs production. But the molecular mechanisms of the effects of liraglutide in the diabetic atherosclerosis are still uncertain. Therefore, we raised the hypothesis that liraglutide could slow the formation and progression of diabetic atherosclerosis via the inhibition of ER stress-induced macrophage apoptosis and MVs production. In the current study, we investigated the role of liraglutide in atherosclerosis in the high fat diet (HFD)/streptozotocin (STZ)-induced diabetic rats. Our results indicate that liraglutide can attenuate diabetic atherosclerosis by inhibiting ER-induced macrophage apoptosis and MVs production.

## Methods

### Animals and experimental protocol

Male Sprague Dawley (SD) rats (Experimental Animal Center of Peking University Health Science Center, Beijing, China), weighting 180–200 g, were studied. The rats were housed in plastic cages on 12 h light–dark cycle at 20–22 °C and humidity 55% ± 5% and fed with a standard chow and tap water ad libitum. The study protocol was approved by the Animal Care and Use Committee of Tianjin Medical University and in accordance with the Guide for the Care and Management of Laboratory Animals. The rats were randomly separated into diabetic model rats (n = 22) and control group rats (n = 8). The former were fed with HFD for 8 weeks and then given tail intravenous injection of STZ (2% STZ at 30 mg/kg, Sigma-Aldrich, USA, dissolved in citrate buffer, pH 4.5, at 4 °C) [14], and the latter were fed with regular chow and injected with the same dose of citrate buffer. After 72 h of the STZ injections, blood samples were harvested from the rat tail vein to check the random blood glucose (RBG). The levels of RBG were measured by glucose oxidase electrode method. The rats with RBG ≥ 16.7 mmol/L were considered to be diabetic rats. The RBG of control group rats are also measured at the same time. HFD/STZ induced diabetic rats were randomly studied in the following two different treated groups, liraglutide group (n = 11) and diabetes group (n = 11). The former were given percutaneous injections of liraglutide (200 μg/kg/d, continuous 12 weeks) [15] and fed with HFD. The latter were given percutaneous injections of the same dose of phosphate buffered saline (PBS) and fed with HFD. The control group rats were given regular chow and PBS at the same time. The levels of fasting blood glucose (FBG) and body weight were measured weekly. At 12 weeks after liraglutide treatment, rats were sacrificed by cervical dislocation under anesthesia with pentobarbital sodium (60 mg/kg intraperitoneal injection). Serums were obtained from the peripheral blood collected by extracting from the inner canthus by centrifugation at 3000 rpm for 10 min for biochemical assays. Aorta specimens were removed carefully after the rats were sacrificed and fixed in formalin for hematoxylin-eosin (HE) and immunohistochemical staining. The other aorta tissues were stored at − 80 °C for performing RT-PCR, western blot and ELISA assays.

### Biochemical assays of peripheral blood

The serum levels of alanine aminotransferase (ALT), aspartate aminotransferase (AST), blood urea nitrogen (BUN), creatinine (Cr), blood glucose, high density lipoprotein-cholesterol (HDL-C), total cholesterol (TC), total triglycerides (TG) and fasting insulin (FINS) were measured using automatic biochemical analyzer (the Hospital of Metabolic Disease of Tianjin Medical University, Tianjin, China). The homeostasis model assessment insulin resistance (HOMA-IR) was calculated by the following formula: HOMA-IR = FBG (mmol/L) × FINS (mIU/L)/22.5.

### HE and immunohistochemical staining

The fixed aorta tissues were embedded in paraffin blocks. 4 μm sections were prepared from paraffin blocks and stained with HE routinely. For cluster of differentiation 68 (CD68) staining, aorta sections were deparaffinized and rehydrated, and then were microwaved at 97 °C for 15 min for antigen retrieval. Tissue sections were placed in 3% hydrogen peroxide for 10 min to quench endogenous peroxidase. Sections were stained for CD68 with a rat anti-CD68 monoclonal antibody (at 1:200 dilution, sc-101447, Santa Cruz, America) and the avidin–biotin–peroxidase complex technique. Diaminobenzidine was applied for final colour development. Terminal deoxynucleotidyl transferase dUTP nick end labeling (TUNEL) staining was performed with the In-Site-Cell-Death-Detection-Kit (Roche, Mannheim) according to the manufacture’s protocol.

### Western blot

The aorta tissues were homogenized in a homogenizer (KIA, T10, German) using sodium dodecyl sulfate polyacrylamide gel electrophoresis (SDS-PAGE) lysis buffer at 4 °C. The homogenates were centrifuged at 12,000*g* for 20 min and the supernatants were boiled in an SDS sample loading buffer for 5 min before electrophoresis on SDS–polyacrylamide gel. Protein concentration was measured using the Bio-Rad protein assay (BioRad, Richmond, USA). After electrophoresis for 1.5 h, proteins in the SDS-PAGE gel were transferred to nitrocellulose membranes at 100 voltage for 2 h. The membranes were blocked in 5% milk for 1 h. Then the membranes were incubated with a primary antibody against β-actin (1:1000, Santa Cruz, USA), caspase-3 (1:1000, Sigma, USA), CHOP (1:1000, Stressgen, USA) or GRP78 (1:1000, Santa Cruz, USA) at 4 °C overnight respectively. After incubating with 1:4000 goat IgG (Santa Cruz, USA) as secondary antibody for 1 h, The membranes were scanned densitometrically by Typhoon (Pharmacia, USA) and quantification of bands was done using Image Total Tech (Pharmacia, USA).

### Quantitative real-time RT-PCR

Total RNA was isolated using Tri Reagent (Sigma-Aldrich). cDNA was synthesized from total RNA with oligo-dT-primers by using a cDNA Kit (Roche) according to the manufacture’s manual. Specific mRNA expressions were quantified using LightCycler Fast Start DNA Master SYBR Green I (Roche). Roche LightCycler software (LightCycler 480 Software Release 1.5.0) was used to perform advanced analysis of relative quantification using the 2^(−ΔΔCt)^ method. Relative gene expressions were given as ×-fold expression of the used housekeeping gene glyceraldehyde 3-phosphate dehydrogenase (GAPDH). Primer sequences for SREBP-1c: forward: 5′TGCTGGACCGCTCCCGCCTG3′, reverse: 5′CTGCTCTCTGCCTCCAGCAT3′, FAS: forward: 5′ATCTGGGCTGTCCTGCCT3′, reverse: 5′GATATAATCCTTCTGAGCAG3′, GAPDH: forward: 5′GGCATTGCTCTCAATGACAA3′, reverse: 5′TGTGAGGGAGATGCTCAGTG3′.

### ELISA of MVs

To quantify the MVs in aorta tissues, the Mircovesicle Assay Kit (#521096, HYPHEN BioMed Company, France) was used according to the manufacturer’s instruction. Aorta tissues were homogenized in a homogenizer using cold normal saline at 4 °C. Then, the homogenates were centrifuged. Aliquots of the supernatants were used for the quantification of MVs. Optical density was measured using the scanning full wavelength spectrophotometer (Thermo, Mk-3, USA).

### Statistical analyses

The data were presented as the mean ± SD. Statistical analyses were performed by using Students *t* test for comparison of two groups and analysis of variance for comparison of multiple groups. A value of *P* < 0.05 was considered to be significant. SPSS 18.0 statistical soft was used for the data analyses.

## Results

### Liraglutide improves the biochemical parameters in T2DM rats

**Fig. 1:**
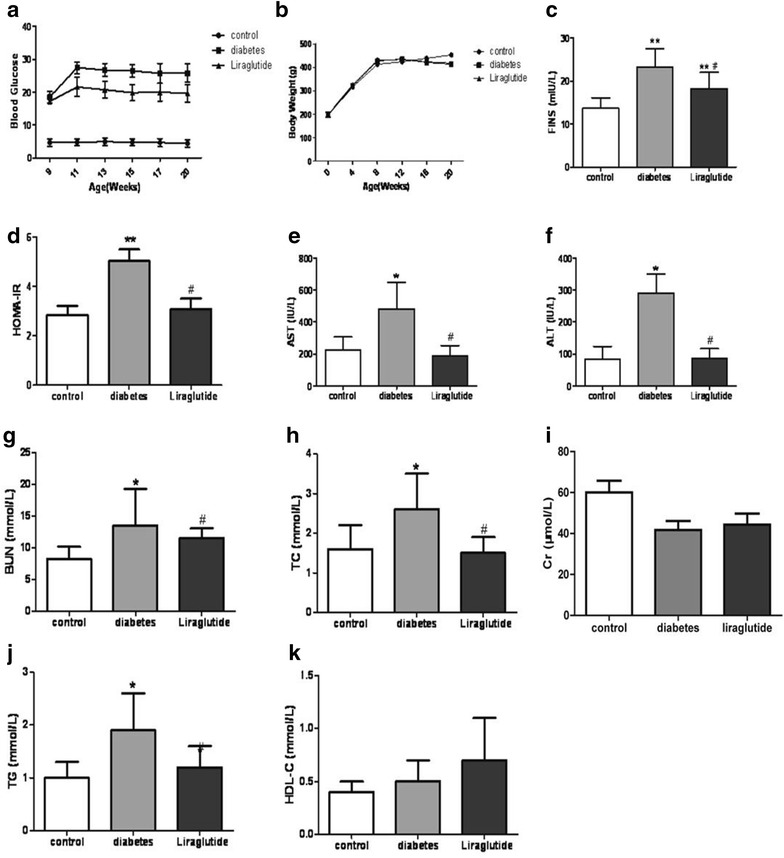
Blood glucose, body weight and biochemical parameters of different groups. n = 8, n = 11 and n = 11 for control group, diabetes group and liraglutide group respectively. Values are mean ± SD. *P < 0.05, **P < 0.01 compared with control group. ^#^ *P* < 0.05, ^##^ *P* < 0.01 compared with diabetes group

During the phase of the experiment, the rats given HFD/STZ displayed increased chow intake, water intake, urination and decreased activity. Moreover, the rats of diabetes group displayed a remarkable mounting of blood glucose level compared with the control group during the latter 12 weeks. However, the rats of liraglutide group exhibited the lower blood glucose level than that in the diabetes group rats. The diabetes group rats also displayed an increasing and then decreasing trend of body weight. Nevertheless, no significant intergroup difference in body weight was observed between diabetes group and liraglutide group. The diabetes group rats displayed significant increases of FINS, HOMA-IR, AST, ALT, BUN, TC and TG levels at the end point compared with the control rats, whereas the rats of liraglutide group displayed significantly lower levels of these parameters compared with the diabetes group. There were no significantly differences in Cr and HDL-C among three groups (Fig. 1).

### Liraglutide alleviates the progression of atherosclerotic plaques

**Fig. 2:**
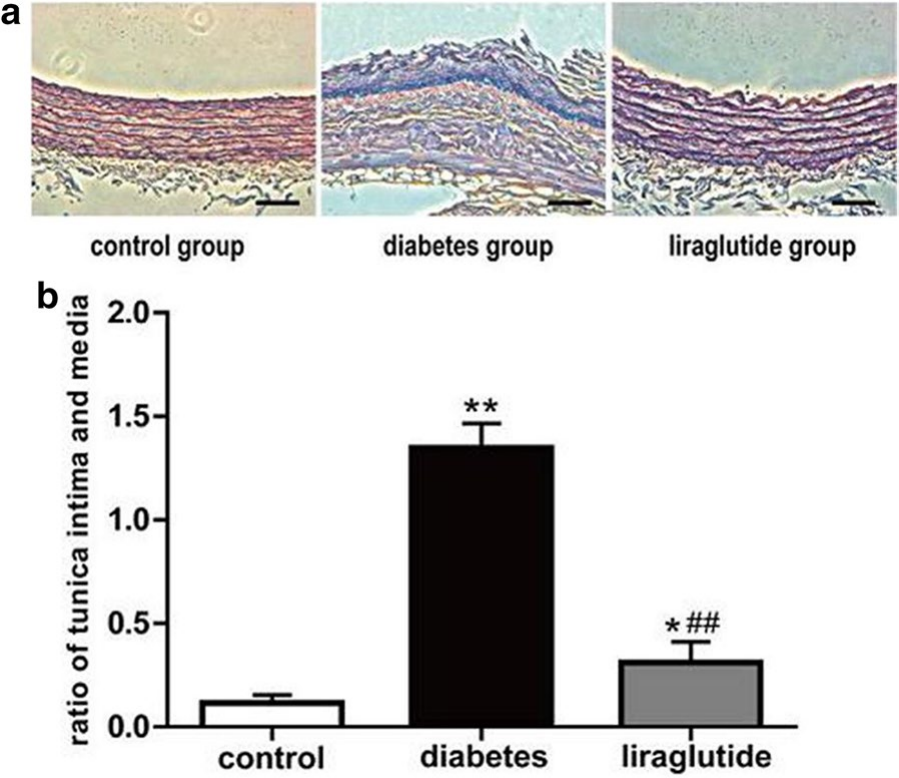
HE staining for aorta and the thickness ratio between aorta tunica intima and tunica media. **a** HE staining. Scale bar: 50 μm. **b** Ratio of tunica intima and media. Values are mean ± SD. n = 8, n = 11 and n = 11 for control group, diabetes group and liraglutide group respectively (**b**). *P < 0.05, **P < 0.01 compared with control group (**b**). ^##^ *P* < 0.01 compared with diabetes group (**b**)

HE staining displayed the apparent atherosclerotic plaques in the aorta tissues of diabetes group rats whereas the normal structure in the tissues of control rats. Moreover, there was the serious destroyed structure of aorta in the diabetes group, including exfoliation of endothelial cells, fracture of elastic fibers, hyperplasia of tunica intima and atrophy of tunica media, however, the slight destroyed changes of aorta histological structure in the rats of liraglutide group (Fig. 2a). There was a higher thickness ratio between tunica intima and tunica media in the diabetes group compared with the liraglutide group (Fig. 2b). Taken together, these data showed that liraglutide played a role in preventing the progression of atherosclerotic plaques.

### Liraglutide inhibits the macrophage apoptosis and microvesicles production

**Fig. 3:**
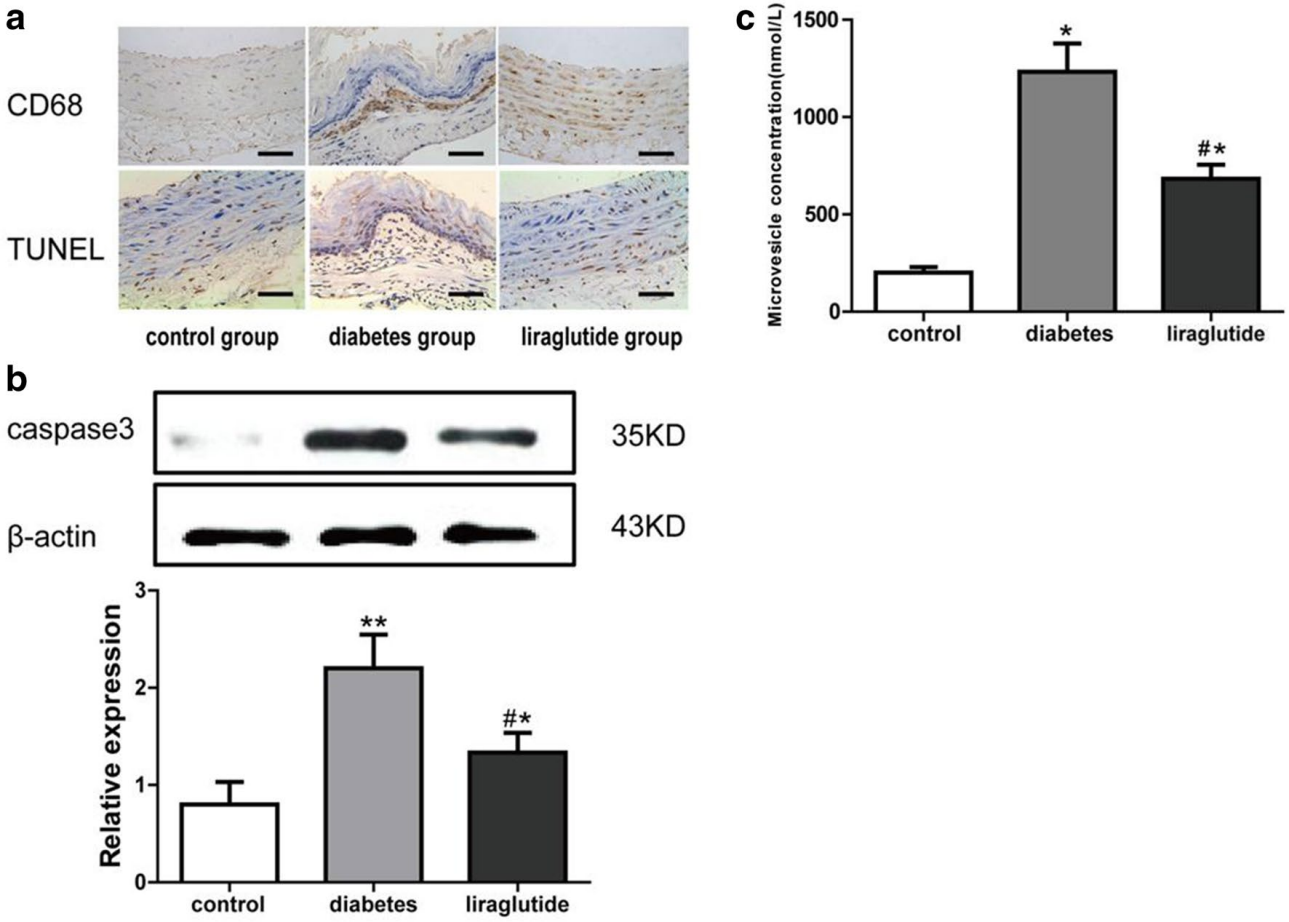
Expression of CD68, TUNEL and caspase-3 and microvesicles concentration in different groups. **a** Immunohistochemistry staining for CD68 and TUNEL. Scale bar: 50 μm. **b** Western blot for caspase-3. **c** Microvesicles concentration. Values are mean ± SD. n = 8, n = 11 and n = 11 for control group, diabetes group and liraglutide group respectively (**b**, **c**). *P < 0.05, **P < 0.01 compared with control group (**b**, **c**). ^#^ *P* < 0.05 compared with diabetes group (**b**, **c**)

Compared with the diabetes group, immunohistochemistry staining showed there was a remarkable decreasing of CD68 positive cells and TUNEL positive cells in the atherosclerotic plaques in the liraglutide group (Fig. 3a). The expression level of caspase-3 tested by western blot in the liraglutide group was obviously lower than that in the diabetes group (Fig. 3b). Moreover, there were significantly fewer MVs in the aorta tissues of liraglutide group than that in the diabetes group (Fig. 3c).

### Liraglutide alleviates the ER stress and inhibits the expressions of lipid metabolism-related factors

**Fig. 4:**
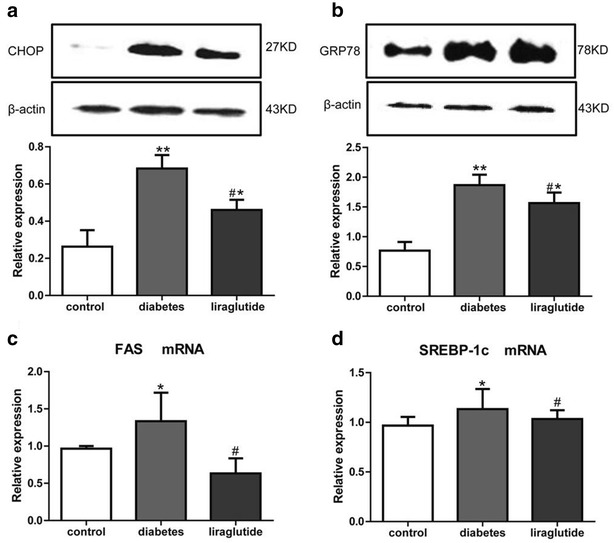
Relatively expression of CHOP, GRP78, SREBP-1c and FAS in different groups. **a** Western blot for CHOP. **b** Western blot for GRP78. **c** Quantitative real-time RT-PCR for mRNA expression of FAS. **d** Quantitative real-time RT-PCR for mRNA expression of SREBP-1c. Values are mean ± SD. n = 8, n = 11 and n = 11 for control group, diabetes group and liraglutide group respectively (**a**, **b**, **c**, **d**). *P < 0.05, **P < 0.01 compared with control group (**a**, **b**, **c**, **d**). ^#^ *P* < 0.05 compared with diabetes group (**a**, **b**, **c**, **d**)

The rats of liraglutide group displayed relatively lower expressions of CHOP and GRP78 which were the marker proteins of ER stress (Fig. 4a, b). Moreover, SREBP-1c and FAS were important relative factors of lipid metabolism. There were dramatic downregulations of FAS mRNA and SREBP-1c in the liraglutide group compared with the diabetes group (Fig. 4c, d).

## Discussion

Atherosclerosis is the pivotal complication for diabetes patients. The current study strongly supports that liraglutide can slow the development of atherosclerosis via inhibiting ER stress-induced macrophage apoptosis and MVs production in T2DM rats. These results manifest that liraglutide is beneficial to diabetes patients against atherosclerosis.

In this study, the diabetic atherosclerosis model has been established successfully in SD rats. These rats have showed a persistent high level of blood glucose and a mounting trend of FINS and HOMA-IR, which displayed that the rats underwent impaired glucose tolerance and diabetes mellitus gradually. The abnormal biochemical variables indicated that the diabetic rats had the liver injury, renal insufficiency and hyperlipoidemia. HE staining of the aortic tissues proved the formation of atherosclerotic plaque in the diabetic rats. This kind of rat model kept stable pathological status, which simulated the progress of atherosclerosis in the diabetic patients. The rat model could be used to estimate the therapeutic effect of liraglutide further.

Liraglutide is currently used for improving glycemic control in the T2DM patients. It also has the beneficial effects on the cardiovascular system [16]. The current study shows liraglutide can reduce the levels of blood glucose, fasting insulin, HOMA-IR, AST, ALT, BUN, TG and TC in the diabetic rats,which is similar to the results of clinical therapy for the diabetic patients [17, 18]. The typical pathological characters of atherosclerosis are lipid accumulation, macrophages infiltration, foam cells forming, formation of plaques and thickening of artery intima. In this study, the thickness ratio between aorta intima and media has been remarkable reduced in the rats with liraglutide treatment. The result is in line with a clinical study which shows that liraglutide can decrease carotid intima thickness and attenuate the formation of atherosclerotic plaques in the patients with T2DM [19]. Macrophages are essential in the development of atherosclerotic plaques. CD68 is looked as a marker of macrophage activation [20]. TUNEL and caspase-3 are the markers of cells apoptosis. In our study, the rats treated with liraglutide have displayed a remarkable decreasing expressions of CD68, TUNEL and caspase-3 in the atherosclerotic plaques. Combined with the results of HE staining, we can get the clues that liraglutide inhibits the macrophages infiltration and apoptosis in the plaques. During the stage of atherosclerosis plaque formation, the defective phagocytic clearance of apoptotic macrophages leads to the secondary inflammatory response and plaque increasing [21, 22]. Accordingly, the progress of atherosclerosis may be suppressed via inhibiting the macrophage apoptosis in the rats with liraglutide treatment. The MVs are produced from the apoptotic cells including the macrophages. Previous study has demonstrated that MVs could initiate the endothelial cells to recruit inflammatory cells and promote the atherosclerotic plaque progress [23]. In the current study, MVs concentration has decreased in the rats of liraglutide group. Therefore we speculate liraglutide can also ameliorate the atherosclerotic plaque by inhibiting MVs production.

Furthermore, we have probed into the mechanisms of inhibiting macrophage apoptosis and MVs production by liraglutide. GRP78 is an important molecular chaperone localized in the ER, which plays a vital role in the recognition of unfolded proteins. CHOP is the downstream protein of the apoptotic pathway and is associated with the ER stress-induced apoptosis [24]. Excessive accumulation of unfolded protein in ER can trigger the cell apoptosis, which is depended on the persistent expression of CHOP [25]. Therefore, GRP78 and CHOP are the important molecular markers of ER stress. These markers are significantly downregulated in the rats of liraglutide group, which shows ER stress is inhibited by liraglutide. Prior study has also shown that liraglutide inhibited the ER stress in β cells of pancreatic islets and played a cell protective effect [26]. On the other hand, liraglutide disturbs the metabolism of lipid via inhibiting ER stress. SREBP-1c coordinates the synthesis of the fatty acid and cholesterol, and FAS is its downstream transcription factor [27]. SREBP-1c can be activated by ER stress and increased the expression in atherosclerosis [28]. The current study has demonstrated the expressions of SREBP-1c mRNA and FAS mRNA were downregulated by liraglutide, which decreased the synthesis and accumulation of lipid in the atherosclerosis plaques. Taken together, these data support that liraglutide attenuates the diabetic atherosclerosis by inhibiting ER stress.

## Conclusion

In conclusion, liraglutide attenuates diabetic atherosclerosis by inhibition of ER stress and subsequent macrophage apoptosis and MVs production. Because ER stress is one of the underlying mechanisms of diabetic atherosclerosis, chronic treatment of liraglutide may significantly contribute to atherosclerosis prevention in diabetes.

## Abbreviations

T2DM: type 2 diabetes mellitus
SD: Sprague–Dawley
ELISA: enzyme-linked immunosorbent assay
CHOP: C/EBP homologous protein
SREBP-1c: sterol regulatory element binding protein-1c
FAS: fatty acid synthase
RT-PCR: reverse transcription polymerase chain reaction
AST: aspartate aminotransferase
ALT: alanine aminotransferase
BUN: blood urea nitrogen
CD68: cluster of differentiation 68
ER: endoplasmic reticulum
MV: microvesicle
GLP-1: glucagon-like peptide-1
HFD: high fat diet
STZ: streptozotocin
RBG: random blood glucose
PBS: phosphate buffered saline
HE: hematoxylin–eosin
Cr: creatinine
HDL-C: high density lipoprotein-cholesterol
TC: total cholesterol
TG: total triglycerides
FINS: fasting insulin
HOMA-IR: homeostasis model assessment insulin resistance
TUNEL: terminal deoxynucleotidyl transferase dUTP nick end labeling
SDS-PAGE: sodium dodecyl sulfate polyacrylamide gel electrophoresis
GAPDH: glyceraldehyde 3-phosphate dehydrogenase
